# Factor Associated with Adherence to the Protein and Fat Counting Strategy by Adults with Type 1 Diabetes Mellitus

**DOI:** 10.3390/nu16121930

**Published:** 2024-06-18

**Authors:** Gabriela Correia Uliana, Juliana Carvalho da Costa, Ayla Rocha Quaresma, Arthur Andrade da Fonseca, Kaory Brito Ohaze, Layla Sandia Cezário Alves, Daniela Lopes Gomes

**Affiliations:** 1 Postgraduate Program in Neurosciences and Behavior, Nucleus of Behavior Theory Research, Federal University of Pará, Belém 66075-110, Brazil; gabriela.ulianafh@gmail.com; 2 Faculty of Nutrition, Federal University of Pará, Belém 66075-110, Brazil; juliana.mjcarvalho@gmail.com (J.C.d.C.); aylaarochaq@gmail.com (A.R.Q.); arthur.adfonseca@gmail.com (A.A.d.F.); kaory456@gmail.com (K.B.O.); laylasandy7@gmail.com (L.S.C.A.)

**Keywords:** strategy, protein and lipid counting, type 1 diabetes mellitus, diet, glycemic control

## Abstract

Carbohydrate counting is one of the dietary strategies used for the management of type 1 diabetes (T1DM), and counting proteins and fats allows individuals to achieve better glycemic and metabolic control, reducing glycemic variability and long-term complications. The aim of this paper is to analyze the factors associated with adherence to the protein- and fat-counting strategy in adults with T1DM. This cross-sectional study was conducted from November 2021 to June 2022 through an online questionnaire. We applied Pearson’s Chi-square test with adjusted residual analysis and a binomial logistic regression test using SPSS software, version 24.0, considering *p* < 0.05 as indicative of statistical significance. There was an association between performing protein and lipid counting and having a higher education level, income exceeding three minimum wages, and having adequate glycated hemoglobin. Performing protein and lipid counting increased the chances of having adequate HbA1c by 4.3 times. Protein and lipid counting was a predictor of having adequate HbA1c. The results suggest that considering the practice of counting proteins and fats is important as a strategy to optimize glycemic control.

## 1. Introduction

Type 1 Diabetes Mellitus (T1DM) is characterized by chronic hyperglycemia caused by insufficient or absent insulin production due to the destruction of pancreatic beta cells [1]. To ensure adherence to treatment, patients need to be disciplined in the various pillars of treatment, such as insulin therapy, regular physical activity, frequent blood glucose monitoring and adherence to healthy habits, as well as being aware of additional strategies that also contribute to glycemic control and help to prevent future complications of the disease [2].

Carbohydrate counting (CC) is one of the most effective strategies for promoting glycemic control in patients with T1DM, as carbohydrate is the macronutrient that most impacts the increase in blood glucose, since it is 100% converted into glucose; this process occurs between fifteen minutes and two hours after food intake [3,4]. However, counting fats and proteins can also be considered beneficial for glycemic and metabolic control, since some studies have shown that these macronutrients can also significantly affect the postprandial glycemic profile [5,6]. According to Paterson et al. [7], it has been shown that there are controversies related to the impact of fat and protein on postprandial glycemia, as the combination of meals composed of the three macronutrients resulted in a reduction in the early postprandial increase and the development of postprandial hyperglycemia in the late period [8].

According to Bozzetto et al. [9], depending on the type of lipid ingested, the glycemic response can be greater or lesser, since there are several mechanisms that influence the glycemic response, one of which is the impact on the action of receptors and hormones [10]. There is evidence that saturated fatty acids worsen postprandial sensitivity and delay gastric emptying, while monounsaturated fatty acids improve postprandial insulin sensitivity and stimulate the secretion of glucagon-like peptide 1, which possibly explains the opposite effects of these types of fats on the glycemic response [9].

In addition, Cutruzzola et al. [11] point out that there are several factors that influence postprandial glucose excursion, since the quantity and quality of macronutrients affect insulin administration and the rate of gastric emptying. In this sense, a meal low in fat and high in fiber showed an increase in postprandial glycemia in one hour, while a meal high in fat and low in fiber reduced glucose concentration by 12% in the same period [12]. In addition, Bozzetto et al. [9] showed that the type of fat has an impact, even if it is not statistically significant, on the postprandial glycemic peak, so that meals with olive oil, a monounsaturated fatty acid, affected early postprandial glycemia 50% less than meals with butter or low saturated fat content. However, it is worth noting that the glycemic index of the meal influences the behavior of blood glucose, regardless of the type and amount of fat added [9]. 

In addition, isolated protein was related to an increase in postprandial glycemia and the consumption of both macronutrients to late hyperglycemia. In these cases, it is necessary to use algorithms that allow these macronutrients to be included in insulin administration [7]. The late increase in blood glucose caused by protein is due to the influence of various hormones and gluconeogenesis, which converts amino acids into glucose [13]. Thus, the adequacy of proteins and fats in relation to insulin doses should also be monitored in patients with T1DM in order to avoid or reduce the risks of late hyperglycemia that can occur as a result of excess or isolated consumption of these nutrients, within a period of two to eight hours after the meal [2].

Bell et al. [14] conducted a study aiming to analyze the impact of fat, protein, and glycemic index on postprandial glucose control in T1DM and the implications related to glucose monitoring. This systematic review utilized database searches and found that all these dietary factors influence postprandial glycemia. The study indicated that meals with a high fat/protein content require more insulin than meals with low fat/protein content, even if both meals have the same carbohydrate content. Pańkowska et al. [5] conducted a randomized study with control groups related to increased postprandial glucose in T1DM patients. It highlighted that, in addition to a sugary meal, meals with proteins and fats also raise blood glucose levels. Both studies identified a higher increase in blood glucose after consuming meals composed of carbohydrates, proteins, and fats compared to meals composed only of carbohydrates.

Proteins are converted into glucose between 30% and 60%, over a period of three to four hours, and fats have 10% of their content converted into glucose over a period of about five hours [3]. Therefore, similar to carbohydrates, though not in equivalent proportions, proteins and fats also trigger changes in glycemic control that can lead to postprandial hyperglycemia [2].

This study aims to analyze the factors associated with the practice of counting proteins and fats by adults with Type 1 Diabetes Mellitus in Brazil. Specifically, it seeks to describe the socioeconomic, demographic, and clinical profile of the participants, identify the prevalence of adherence to the practice of counting proteins and fats in the studied population, characterize the nutritional status and self-reported glycemic control of the participants, analyze the variables associated with the practice of counting proteins and fats, and examine how these factors are associated and impact the outcomes of those who adhere to the macronutrient counting strategy in question. Our hypothesis is that participants who counted proteins and lipids had better glycemic control.

## 2. Materials and Methods

### 2.1. Type of Study

This exploratory study was conducted from November 2021 to June 2022. The study was disseminated through the social media platforms (WhatsApp^®^, Facebook^®^, and Instagram^®^) of the researchers and the Extension Project “Educational Group in Diabetes”, in affiliation with the nutrition faculty of a public university in the northern region of Brazil. It was directly sent to individuals who declared having Type 1 Diabetes (T1DM) in their social media biographies. This work is part of a larger research project titled “Factors associated with difficulties in adhering to carbohydrate counting by adults with Type 1 Diabetes Mellitus”.

### 2.2. Participants

Convenience sampling was used with adults with T1DM residing in Brazil who agreed to the Informed Consent Form (ICF). Participants could be of either sex, but needed to have a diagnosis of T1DM, be aged between 18 and 59, have knowledge about carbohydrate counting strategy, and consent to the ICF from the Federal University of Pará, under approval number 5.077.488.

### 2.3. Instrument

An online form, built on the Google Forms^®^ platform, was used in a survey format following Resolution 510 of 7 April 2016 [10]. The original survey form contained 65 questions, with 60 being objective and 5 being simple subjective questions (weight, height, HbA1c value, age, and diagnosis time in years). However, for this study, only 23 objective and 5 subjective questions were used. The questions were divided into four axes, namely:

1. Carbohydrate counting knowledge: This section includes questions related to knowledge of carbohydrate counting (CC), such as when CC is performed, the method used to determine the quantity of carbohydrates in foods, whether a kitchen scale is used for CC, and the reasons for using a kitchen scale.
2. Clinical and anthropometric data: This section contains questions related to Body Mass Index (BMI), BMI classification, HbA1c examination, the value of HbA1c in the participants’ last examination, and the duration of T1DM diagnosis.
3. Sociodemographic and socioeconomic: This section includes questions about age, biological sex, place of residence (state, city, and neighborhood), level of education, and family income.
4. Health professionals’ follow-up (considering the three months prior to the survey): These questions cover multiprofessional assistance, whether participants have followed up with any healthcare professional, the mode of attendance (in-person, virtual, both, or none), the method of follow-up (through health insurance, public health system, both, or private), whether participants perform protein and lipid counting, who taught them to perform protein and lipid counting, and when they perform protein and lipid counting.

### 2.4. Data Analysis

Data collection occurred online through the dissemination of the research via a link. Upon clicking the provided link, individuals were directed to a brief explanation of the study. Subsequently, a link to the Informed Consent Form (ICF) was provided, specifying that no participant identification was necessary. Participants could choose to agree or decline to participate in the research. If they selected the option “I do not agree to participate in the research”, the survey was automatically terminated. If they chose the option “I have read the ICF and agree to participate in the research”, the participant was directed to a page where another inclusion criterion was applied.

Next, questions related to each axis were presented: (1) carbohydrate counting knowledge; (2) clinical and anthropometric data; (3) sociodemographic and economic; and (4) health professional follow-up. On the last page of the survey, a link was provided for access to the Carbohydrate Counting Manual for People with Diabetes [9].

For statistical analysis, the Statistical Package for Social Science, version 24.0, was utilized. Descriptive results were presented in absolute frequency and proportion. In the analytical phase, Pearson’s Chi-square test with adjusted residual analysis was applied to identify associated categories. The statistical significance level considered was *p* < 0.05.

## 3. Results

The socioeconomic and demographic characterization of this population sample has been previously published in the work of Uliana et al. [14]. The characteristics regarding protein and lipid counting in a sample of 173 adult individuals with T1DM in Brazil in 2020 were examined. Predominantly, respondents were aware of what protein and lipid counting is but did not know how to perform such a count (32.4%), while 11.6% knew how but had never attempted it; 12.7% had performed protein and lipid counting in the past but had abandoned the practice, 20.8% did not know what protein and lipid counting is, and 22.5% were currently practicing counting.

Regarding the healthcare professional who taught protein and/or lipid counting, the majority received assistance from a nutritionist (26.0%), while 11.6% were assisted by an endocrinologist. The study assessed during which meals protein and/or lipid counting was performed, and it was observed that it predominantly occurred during main meals such as dinner (23.7%), lunch (22.5%), and breakfast (15.0%). Only 11.0% of people counted proteins and lipids during their morning snack, 11.0% during the afternoon snack, and 11.6% during supper (Table 1).

When analyzing associations between socioeconomic data and the practice of protein and fat counting, no association was found between performing or not performing protein and fat counting and biological sex. However, performing protein and lipid counting was associated with having a higher education level (*p* = 0.002) and income exceeding three minimum wages (*p* = 0.002) (Table 2).

In relation to protein and lipid counting and clinical and nutritional data, it was observed that performing protein and lipid counting was associated with having adequate HbA1c (*p* < 0.0001) (Table 3).

No associations were found between the practice of protein and lipid counting and the duration of diagnosis, the consultation model used in recent months, and follow-up with a healthcare professional (Table 3).

Prior to conducting logistic regression analysis, the absence of collinearity between study variables was verified through linear regression. The tolerance and VIF values were all greater than 0.1 and less than 10, respectively. Subsequently, binomial logistic regression analysis was performed, with adequacy of HbA1c (adequate or inadequate) according to reference values as the dependent variable and the practice of counting proteins and lipids (yes or no) and having learned to count proteins and lipids with a nutritionist (yes or no) as the independent variable. The final model predicted 67% adequacy in the studied sample. 

Performing protein and lipid counts and having learned how to count proteins and lipids from a nutritionist were significant predictors of adequate HbA1c. Participants who learned protein and lipid counting from a nutritionist had 3.6 times higher chances of having adequate HbA1c. Similarly, participants who performed protein and lipid counting had 2.6 times more chances of having adequate HbA1c (Table 4).

## 4. Discussion

In the present study, we observed that a large percentage of respondents knew what protein and lipid counting was but did not know how to perform such a count themselves (32.4%), which is a significantly higher proportion compared to what was observed in the study by Uliana et al. [15]. This previous study investigated knowledge about CC, where participants were aware of what CC was but did not know how to perform it (18.01%). Therefore, it is suggested that individuals with T1DM have more knowledge about CC than about protein and lipid counting. It is also possible that the method of protein and lipid counting is less well known than CC, likely due to its less frequent usage, leading to lower dissemination as a nutritional strategy.

Furthermore, it was observed that the healthcare professional who most often taught respondents about protein and lipid counting was the nutritionist (26.0%). It is worth noting that no studies were found that specifically addressed the contribution of nutritionists in teaching protein and lipid counting. However, in the study by Souza et al. [16], which focused on identifying and discussing the knowledge of professionals and patients regarding CC and the nutritionist’s involvement in its execution, it was observed that the nutritionist’s role is crucial in the education process for T1DM due to their more in-depth knowledge of food composition. In a similar context, the study conducted by Gabriel et al. [17], which aimed to develop and evaluate the effectiveness of an educational program for empowering adolescents with T1DM to count carbohydrates without parental assistance, highlighted the nutritionist as the main professional who conducted nutrition education activities focused on CC. Thus, there is evidence that the most qualified professional for promoting nutrition education in protein and lipid counting is the nutritionist. However, it is important to emphasize the ongoing need for better training for nutritionists in the field of macronutrient counting, which is crucial in the nutritional care of DM patients.

It was also observed that protein and lipid counting predominantly occurred during meals such as lunch and dinner. It is presumed that, as these are meals individuals typically have at specific times, where they have more available time to carry them out, there is more time to use the strategy of protein and lipid counting. In a study by Uliana et al. [18], it was highlighted that a higher frequency of meals among people with T1DM was associated with not performing CC. Similar to CC, protein and lipid counting require the ability to identify the quantity of the macronutrient present in the food that makes up the meal [3,19]. In this regard, it is suggested that these criteria also be applied to the execution of protein and lipid counting, given that prior knowledge of CC is recommended for using this strategy. Additionally, in general, meals like lunch and dinner have a higher proportion of proteins and lipids in their composition, which may motivate patients to apply protein and lipid counting preferably during these meals.

Having a higher education level and an income greater than three minimum wages were associated with performing protein and lipid counting. Ewers et al. [20] state that calculations requiring good mathematical knowledge are necessary for CC. Furthermore, in the study by Uliana et al. [18], not performing CC was associated with not having a higher education degree. In this sense, it is suggested that both CC and protein and lipid counting require patients to have more specific knowledge and mathematical skills to perform the necessary calculations. Additionally, the same author describes that supplies for blood glucose self-monitoring are needed, and these are not always provided by the public system in sufficient quantities for adequate monitoring, requiring some individuals to bear the costs of acquiring these supplies. Thus, the hypothesis is raised that having an income greater than three minimum wages favors adherence to the protein and lipid counting strategy in an adequate manner.

In this study, we also found an association between the practice of protein and lipid counting and having adequate HbA1c, where 15.6% of individuals who performed protein and lipid counting had adequate HbA1c. In the study by Paterson et al. [13], the impact of these macronutrients on postprandial blood glucose is evident; in the case of lipids and proteins, when consumed together, their effects on blood glucose accumulate, leading to a higher postprandial glycemic response. HbA1c reflects the average levels of blood glucose over the past three or four months; thus, regular blood glucose monitoring and HbA1c measurement are complementary tools in glycemic control [2,21,22].

In this work, practicing protein and lipid counting was a significant predictor for having adequate HbA1c. In the study conducted by Uliana et al. [18], it was demonstrated that practicing CC also predicts HbA1c adequacy, both being independent of the time of diagnosis. In this sense, it is suggested that adherence to the macronutrient counting strategy is related to an improvement in glycemic control. Furthermore, it is worth noting that individuals practicing protein and lipid counting had 2.6 times greater chances of having adequate HbA1c, and those practicing CC demonstrated 3.273 times the chances of HbA1c adequacy.

While carbohydrates traditionally receive priority attention, the SBD [2] reports that including proteins and lipids in the scenario reveals a more comprehensive dietary landscape that directly impacts metabolic health. Examining these interactions highlights the need for integrated approaches in patient treatment, where counting all components of the diet—proteins, carbohydrates, and lipids—is crucial for promoting optimal HbA1c levels and, by extension, enduring metabolic health.

This study faced the challenge of a scarcity of research addressing the relationship between protein and lipid counting and T1DM for use in comparing results and discussing key findings. Scientific literature often emphasizes the investigation of traditional variables such as blood glucose, CC, and HbA1c, relegating the exploration of interactions between these biomarkers and the protein and lipid components of metabolism to a secondary role. 

The limitation in the availability of studies focused on this association made it difficult to obtain robust evidence to underpin our discussion. The present research has some limitations, such as using self-reported values for weight, height, duration of diagnosis, and HbA1c. Additionally, being an online survey, it only included participants with internet access. 

It is crucial to recognize that the absence of a substantial volume of studies in this field indicates the need for future research to provide a better understanding of the interactions between glycemic parameters and the consumption of proteins and lipids for people with T1DM, especially.

## 5. Conclusions

It is concluded that the majority of respondents knew what protein and lipid counting was but did not know how to perform it, highlighting the need to invest in diabetes education. It was also observed that factors associated with greater adherence to protein and lipid counting included receiving guidance from a nutritionist, having a family income greater than three minimum wages, having a higher education level, and having free time during meal preparation. Furthermore, this study demonstrated that adhering to protein and lipid counting was associated with adequate HbA1c control. These results emphasize the importance of considering protein and lipid counting as a strategy to optimize glycemic control. In addition, this study can contribute information on aspects associated with adherence to the protein and lipid counting strategy to differentiate patients who may be more successful with the implementation of this strategy.

## Figures and Tables

**Table 1:** Characterization of the practice of protein and fat counting by adults with DM1 in Brazil, 2020.

	n	%
Adherence to Protein and/or Lipid Counting		
I’ve done Protein and/or Lipid Counting for a while, but I’m not doing it at the moment	22	12.6
Yes, I do the Protein and/or Lipid Count	39	22.5
I know how to do it, but I’ve never done a Protein and/or Lipid Count	20	11.6
I know what Protein and/or Lipid Counting is, but I don’t know how to do it	56	32.4
I don’t know what Protein and/or Lipid Count is	36	20.8
Health professional who taught Protein and/or Lipid Counting		
Endocrinologist	20	11.6
Nutritionist	45	26
Protein and/or lipid counting at mealtimes		
Breakfast	26	15.0
Morning snack	19	11.0
Lunch	39	22.5
Afternoon snack	19	11.0
Dinner	41	23.7
Supper	20	11.6

**Table 2:** Association between protein and lipid counts and socioeconomic data of adults with DM1 in Brazil in 2020.

	Protein and Lipid Count		*p*-Value *
	Yes n (%)	No n (%)	
Education			
No higher education	10 (5.8) (−)	72 (41.6) (+)	0.002 ^†^
With higher education	29 (16.8) (+)	62 (35.8) (−)	
Family income			
Up to 3 minimum wages	9 (5.2) (−)	68 (39.3) (+)	0.002 ^†^
More than 3 minimum wages	30 (17.3) (+)	66 (38.2) (−)	
Biological sex			
Male	3 (1.7)	24 (13.8)	0.122
Female	36 (20.8)	110 (63.6)	

* Chi-square/Fisher exact. † Statistical significance; analysis of residuals: (+) positive significant association; (−) negative significant association; minimum wage = BRL 1.100.

**Table 3:** Association between protein and lipid counts and clinical and nutritional data of adults with DM1 in Brazil in 2020.

	Protein and Lipid Count		*p*-Value *
	Yes n (%)	No n (%)	
BMI classification			
Adequate	27 (15.6)	77 (44.5)	0.187
Not adequate	12 (6.9)	57 (32.8)	
HbA1c classification			
Adequate	28 (15.6) (+)	47 (27.2)	<0.0001 ^†^
Increased	12 (6.9)	87 (50.3)	
Diagnostic time			
<10 years	12 (6.9)	37 (21.4)	0.700
>10 years	27 (15.6)	97 (56.1)	
Accompanied by an endocrinologist			
Yes	37 (21.4)	130 (75.1)	0.520
No	2 (1.2)	4 (2.3)	
Accompanied by a nutritionist			
Yes	19 (11.0)	55 (31.8)	0.394
No	20 (11.6)	79 (45.7)	
Consultation in recent months			
In person	25 (14.5)	75 (43.4)	0.079
Via the internet	4 (2.3)	5 (2.9)	
In person and online	7 (4.0)	22 (12.7)	
No appointments	3 (1.7)	32 (18.5)	

* Chi-Square. ^†^ Statistical significance; analysis of residuals: (+) significant association; BMI = Body Mass Index; HbA1c = glycated hemoglobin.

**Table 4:** Binary logistic regression between HbA1c adequacy and adherence to protein and lipid counting and having learned to counting proteins and lipids with a nutritionist.

	B	S.E.	Wald	*df*	Sig.	EXP (B)	95% C.I. for EXP (B)	
							Lower	Upper
Adherence to protein and lipid counting	0.967	0.425	5.178	1	0.023	2.630	1.143	6.047
Having learned to counting proteins and lipids with a nutritionist	1.306	0.403	10.520	1	0.001	3.692	1.677	8.131
Constant	−3.696	0.899	16.892	1	0.000	0.025		

Binomial logistic regression. Dependent variable: adequacy of glycated hemoglobin; independent variable: adherence to protein and lipid counting (yes or no) and having learned to count proteins and lipids with a nutritionist (yes or no). EXP (B) is OR—odds ratio (OR = eb); Sig. = Statistical significance; S.E. = standard error; B= regression coefficient.

## Data Availability

The data presented in this study are available on request from the corresponding author. The data are not publicly available due to the research were obtained through an online form that allows access to other data not used in this article.
